# Mucosal Schwann Cell Hamartoma of the Appendix: Expanding the Differential for Gastrointestinal Bleeding

**DOI:** 10.14309/crj.0000000000001708

**Published:** 2025-05-21

**Authors:** Abdulmalik Saleem, Taher Jamali, Iana Gueorguieva

**Affiliations:** 1 Department of Internal Medicine, Henry Ford Hospital, Detroit, MI; 2 Department of Gastroenterology and Hepatology, Henry Ford Hospital, Detroit, MI

**Keywords:** Mucosal Schwann Cell Hamartoma, appendix, gastrointestinal bleeding, spindle cells, appendiceal mass

## Abstract

Mucosal Schwann cell hamartomas (MSCH) are rare benign tumors typically found in the colorectal region. We present the case of an 87-year-old man with a history of mucosa-associated lymphoid tissue lymphoma who developed symptomatic gastrointestinal bleeding due to an appendiceal MSCH. The patient's ongoing melena and anemia led to further evaluation. Subsequent colonoscopy revealed active bleeding from the appendiceal orifice with hemostasis achieved using epinephrine injection. A laparoscopic appendectomy confirmed MSCH through histopathology and immunohistochemical staining. Given its rarity, this case underscores the importance of considering MSCH in unexplained gastrointestinal bleeding and demonstrates its potential to be a symptomatically significant entity.

## INTRODUCTION

Mucosal Schwann cell hamartomas (MSCH) are benign tumors consisting of mesenchymal cells within the lamina propria of the gastrointestinal tract.^1^ These lesions predominantly occur in the distal colon, particularly the sigmoid, and often present as small, solitary, sessile polyps.^1^ Given their rarity, the literature discussing their clinical presentation, management, and surveillance is scarce.^2–4^ Here, we present the first rare case of MSCH discovered in the appendix of a symptomatic patient.

## CASE REPORT

An 87-year-old White man with a history of mucosa-associated lymphoid tissue (MALT) lymphoma (diagnosed 12 years prior), hypertension, and atrial fibrillation (not on anticoagulation) presented with a 2-day history of abdominal pain and melena. His MALT lymphoma was treated with 4 weeks of rituximab, with an appropriate response and ongoing serial monitoring. The patient had no family history of gastrointestinal cancer, familial adenomatous polyposis, or Cowden syndrome.

On presentation, he was hemodynamically stable. Digital rectal examination revealed melena. Laboratory results demonstrated a hemoglobin drop of 5 g/dL over a 2-month period, necessitating transfusion of 2 units of packed red blood cells, with an appropriate response. His medical history included diverticulosis without active bleeding, and an esophagogastroduodenoscopy 3 years prior that had revealed a large, nonbleeding ulcer.

**Figure 1.**
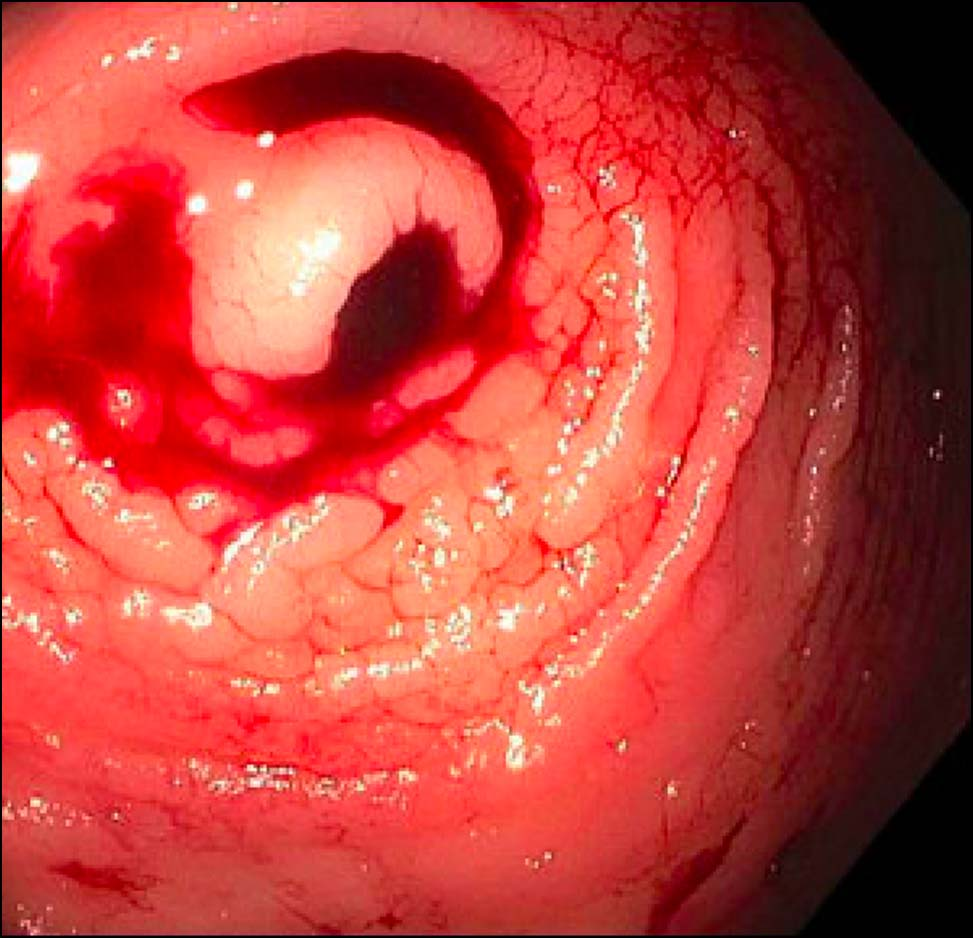
Bleeding notes from appendiceal orifice on colonoscopy.

The patient was admitted for further gastrointestinal evaluation. Esophagogastroduodenoscopy demonstrated a 10-mm healed ulcer in the proximal gastric body and a short segment of Barrett esophagus. Computed tomography of the abdomen and pelvis with intravenous contrast revealed no intestinal or appendiceal pathology. Colonoscopy showed abnormal-appearing rectal tissue, blood throughout the examined colon, and active bleeding from the appendiceal orifice (Figure 1). Hemostasis was successfully achieved with epinephrine injection around the bleeding orifice. His hemoglobin remained stable until laparoscopic appendectomy was performed during the admission for definitive management of the lesion. Intraoperative gross examination demonstrated a normal appearing appendix with a prominent tip without evidence of perforation or obvious masses.

**Figure 2.**
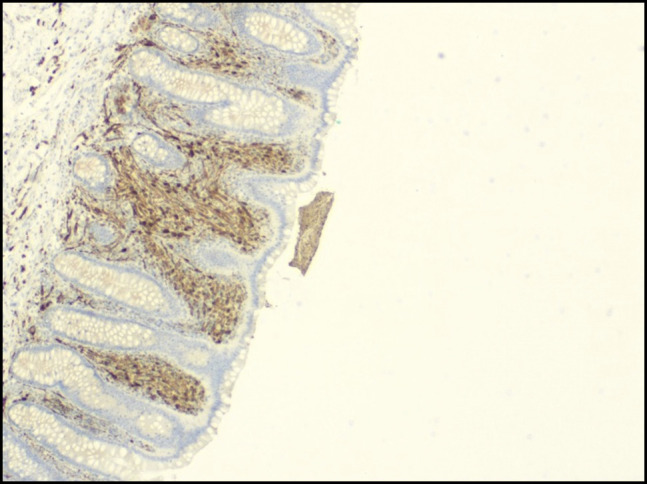
Immunohistochemistry for S-100 (4×).

**Figure 3.**
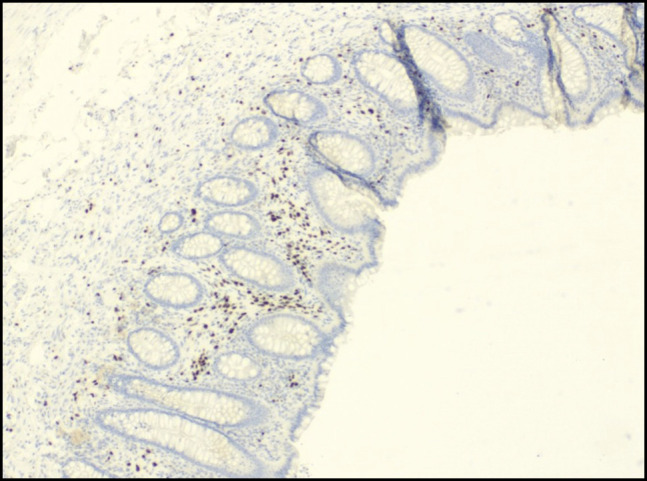
Immunohistochemistry for SRY-related HMG-box gene 10 (4×).

Histopathologic examination of the appendix revealed focal lamina propria spindle cell proliferation. Immunohistochemical staining demonstrated spindle cells positive for S-100 protein and SRY-related HMG-box gene 10, while negative for C-kit, discovered on GIST-1, desmin, smooth muscle actin, epithelial membrane antigen, glucose transporter type 1, Melan-A, and pancytokeratin anti-epithelial antibody 1/anti-epithelial antibody 3 (Figures 2 and 3). These findings were consistent with a benign mucosal Schwann cell hamartoma. The patient had an uneventful postoperative course and remains asymptomatic on follow-up.

## DISCUSSION

MSCH are characterized by a diffuse proliferation of S-100 positive spindle cells within the lamina propria and absence of ganglion cells, distinguishing these tumors from other neural lesions such as neurofibromas or mucosal neuromas.^1,3,5^ Staining is positive for S-100, with one other known documented case of positive for SRY-related HMG-box gene 10.^6^ Although MSCH shares similarities with Schwannoma, as both are composed of spindle cells, they can be differentiated histologically. Schwannomas typically display spindle cells arranged in Antoni A and Antoni B patterns and are encapsulated, whereas MSCH exhibit a diffuse, nonencapsulated growth pattern in the lamina propria.^7,8^

This pathology is exceedingly rare, with fewer than 100 reported cases in the literature. Our case is particularly unique for several reasons. First, although MSCH has been primarily described in the colorectal region—especially the rectosigmoid—it has also been reported in the gastroesophageal junction, gastric antrum, and gallbladder.^1,9–11^ However, to the best of our knowledge, this is the first reported case of MSCH in the appendix. Appendiceal tumors are typically neuroendocrine or epithelial in origin, making this an unexpected finding.^1^

Second, MSCH are typically asymptomatic and detected incidentally during routine screening. Most MSCH are reported asymptomatic in the literature; however, less frequent presentations depending on anatomic region have reported abdominal pain, positive fecal occult blood, or tenesmus (Table 1).^1–6,12–25^ Cases found in the right colon were asymptomatic; however, our patient had the unique presentation of symptomatic anemia and abdominal pain due to a right-sided lesion.

**Table 1.** Clinical characteristics of reported cases of MSCH

Reference	No. of cases	Age (yr)	Sex	Symptom	Location	Gross appearance
Gibson and Hornick^3^	26	Mean 62	M:F 10:16	Asymptomatic, diarrhea, lower GIB	Primarily in rectosigmoid	Sessile polyps, (1–6 mm); mean 2.5 mm
Pasquini et al^5^	1	60	F	Positive fecal occult blood	Rectosigmoid	5-mm sessile polyp
Rocco et al^12^	1	67	F	Asymptomatic	Sigmoid colon	3-mm sessile polyp
Sagami et al^13^	1	40	F	Positive fecal occult blood	Sigmoid	Small, whitish nodules
Bae et al^14^	1	41	F	Asymptomatic	Descending colon	8-mm polyp
Neis et al^15^	1	59	M	Underlying uc	Sigmoid	3-mm polyp
Beca et al^16^	1	72	M	Asymptomatic	Sigmoid	5-mm polyp
Klair et al^17^	1	78	F	Abdominal pain, tenesmus	Rectum	7-mm rectal polyp
Bae et al^18^	1	20	M	Loose stools, abdominal discomfort	Rectum	4-mm polypoid mucosal elevation
Kanar et al^19^	1	67	M	Asymptomatic	Sigmoid	6-mm polyp
Han et al^20^	1	49	M	Asymptomatic	Rectum	2-mm polyp
Gaspar et al^21^	1	42	M	Rectal bleeding	Rectum	30 × 15-mm flat granular lesion
Chintanaboina and Clarke^2^	1	55	F	Asymptomatic	Ascending colon	5-mm tubular adenoma
Hashimoto et al^22^	1	40	F	Positive fecal occult blood	Sigmoid colon	5-mm sessile polyp
Lorenzo et al^23^	1	54	M	Positive fecal occult blood	15 cm from anal margin	5-mm sessile polyp
Okamoto et al^1^	1	64	M	Asymptomatic	Sigmoid colon	Submucosal tumor like protrusions
Barreiro et al^4^	1	50	F	Painless bleeding	Rectum	Erythematous indurated mucosa
Altaf et al^24^	1	48	F	Asymptomatic	Sigmoid	3-mm polyp
Barjas et al^6^	1	60	M	Asymptomatic	Transverse colon	2-mm sessile polyp
Ucar et al^25^	1	65	F	Asymptomatic	30 cm from external anal margin	3-mm polyp

GIB,  gastrointestinal bleeding; MSCH, Mucosal Schwann cell hamartomas.

Third, MSCH typically present endoscopically as a small, sessile, polypoid lesion (Okamoto).^1^ However, in our case, the lesion was identified solely through active bleeding at the appendiceal orifice, without a discrete polypoid mass noted on endoscopy.

Finally, MSCH are more commonly reported in middle-aged women, whereas our patient was an elderly male, further distinguishing this case from previously described presentations.^3^

Risk stratification of MSCH remains challenging as it is not associated with hereditary genetic disorders or other predisposing medical conditions.^3^ The patient's history of MALT lymphoma and rituximab treatment would not have increased his risk for developing neoplastic lesions, either benign or malignant.^26,27^

This case presented a diagnostic challenge as imaging was unable to successfully localize the lesion or source of bleed. Similarly, colonoscopy did not reveal identifiable ulceration or mucosal abnormality involving the appendix. Pathologic diagnosis was essential for this case given the overlap of MSCH with other spindle cell neoplasms, particularly schwannomas.^7^ Moreover, given the grossly normal appearance of the appendix on colonoscopy and intraoperatively, endoscopic ultrasound could have been considered as part of initial evaluation to further characterize layer of origin, size, and echogenicity.^28^

Previously reported presentations of MSCH are summarized in Table 1. Our unusual presentation emphasizes the importance of maintaining a broad differential diagnosis for gastrointestinal bleeding, particularly when classic sources such as the stomach, duodenum, and colon have been ruled out.

Surgical resection remains the definitive treatment for symptomatic appendiceal masses, including hamartomas, following temporizing nonoperative measures.^29^ In this case, endoscopic epinephrine injection effectively stabilized the bleeding, allowing for subsequent laparoscopic appendectomy. Successful resection not only resolved the patient's symptoms but also provided a definitive diagnosis, emphasizing the role of surgical intervention in both therapeutic and diagnostic management of symptomatic cases.

MSCH are a benign pathology with no reported evidence of malignant transformation.^3^ However, the symptomatic presentation in our patient, combined with the overall rarity of this lesion, stresses the need for further case reports and studies to better define its clinical presentation and optimal management. Currently, there are no formal surveillance guidelines for MSCH. However, surveillance may not be required, as a review by Gibson et al reported no recurrences in 26 cases with a mean follow-up of 6.5 years.^3^

With the variable presentation of MSCH, each additional report contributes valuable data regarding natural history, recurrence risk, and long-term outcomes and can further refine risk assessment and guide clinical decision making. Advanced imaging modalities such as endoscopic ultrasound can be valuable in characterizing these submucosal lesions and should be explored. No inherited syndromes have been linked with this pathology; further reports and studies may allow us to clarify a distinctive genetic mutational profile for MSCH.

## DISCLOSURES

Author contributions: T. Jamali and A. Saleem: conceptualization, data curation, formal analysis, methodology, writing—original draft, writing—review and editing, supervision, final approval of the version to be published, accountability for all aspects of the work. I. Gueorguieva: conceptualization, data curation, formal analysis, methodology, writing—review and editing, final approval of the version to be published, accountability for all aspects of the work. A. Saleem is the article guarantor.

Financial disclosure: None to report.

Informed consent was obtained for this case report.
